# EHMTI-0161. Personality profiles of patients with chronic post-traumatic headache: a case-control study

**DOI:** 10.1186/1129-2377-15-S1-C30

**Published:** 2014-09-18

**Authors:** D Kjeldgaard Nielsen, H Forchhammer, TW Teasdale, RH Jensen

**Affiliations:** 1 Department of Neurology Glostrup Hospital, Danish Headache Center University of Copenhagen, Glostrup, Denmark; 2 Department of Neurology Glostrup Hospital, University of Copenhagen, Glostrup, Denmark; 3 The Department of Psychology, Faculty of Social Sciences University of Copenhagen, Copenhagen, Denmark

## Background

The aetiology of chronic post-traumatic headache (CPTH) after mild head injury is unclear, but psychological stress and personality factors have been suggested as both causing and maintaining factors. Research is scarce and it is uncertain whether patients with CPTH share a specific personality profile and if the profile differ from other chronic headache patients.

## Aim

To investigate whether CPTH is distinguished from other chronic headache patients in terms of mental distress, general personality characteristics and traits specifically as measured by the The Revised NEO Personality Inventory (NEO-PI-R).

## Methods

Ninety patients with CPTH and 45 control patients with chronic primary headaches were enrolled from the Danish Headache Center. They completed NEO-PI-R personality assessment, The Symptom Checklist (SCL-90-R) and a headache diary for 4 weeks.

## Results

The scale scores and the levels of psychological distress did not differ between the CPTH and the headache control group. CPTH patients with a headache duration over two years had a higher score on the domain Neuroticism (p=0.002), but lower Extraversion (p=0.001) and lower Openness (p=0.038) on the NEO-PI-R.

## Conclusion

In contrast to prior hypothesis patients with CPTH were not characterised by a specific personality profile although long lasting CPTH was associated to higher neuroticism and a lower degree of extraversion and openness. No differences were found in the reported level of psychological distress in patients with CPTH compared to a headache control group.

No conflict of interest.

